# Molecular Engineering of Carboxylated Polysulfone Membranes for Enhancing Salt Rejection

**DOI:** 10.3390/polym17131840

**Published:** 2025-06-30

**Authors:** Zhuonan Chen, Moris S. Eisen

**Affiliations:** 1 Schulich Faculty of Chemistry, Technion-Israel Institute of Technology, Haifa 32000, Israel; chen.zhuonan@campus.technion.ac.il; 2 Department of Chemistry, Guangdong Technion-Israel Institute of Technology, Shantou 515063, China

**Keywords:** carboxylated polysulfone membranes, crosslinking, brush membranes, pore formation, biofilm prevention

## Abstract

Developing advanced polysulfone (PSF) membranes for water treatment has garnered significant attention. However, carboxylated polysulfone membranes have shown limited rejection of various ions. This study explores four novel methods for modifying carboxylated polysulfone membranes to enhance their performance: (a) crosslinking of the dicarboxylated membrane surface with polyethylenimine or ethylenediamine, (b) partial hydrolysis of ethylenediamine-crosslinked dicarboxylated membranes to create tailored pores and surface brushes with terminal amine groups, (c) attachment of alkyl brushes to the dicarboxylated membrane surface, and (d) formation of quaternary ammonium moieties at the membrane surface. All modified membranes were fully characterized, and their enhanced functionality was confirmed. For instance, the PSF-PEI membrane exhibited a 28% CaCl_2_ rejection and PSF-NH_2_ showed improved CaCl_2_ rejection up to 37%, compared to 0% for the unmodified PSF-COOH. These methods present practical strategies to modify carboxylated-related membranes further, offering potential pathways to enhance their performance.

## 1. Introduction

There has been a significant focus on new and market-oriented applications of polymers due to their varied chemical properties, widespread availability, ease of processing, and expected durability for a wide range of uses [1,2,3,4,5]. Among these, polysulfone (PSF) has been extensively studied and remains a popular material due to its unique combination of exceptional chemical stability, high mechanical strength, inherent surface charge, and broad operational range across different temperatures and pH levels [6,7,8,9,10,11]. These advantageous properties render PSF suitable for water treatment processes when used in pressure-driven filtrations [12,13]. However, PSF’s inherent hydrophobic nature limits its efficacy in water treatment applications. Therefore, the structure of PSF must continuously be modified to achieve more favorable features and overcome this limitations [14,15].

Among various post-modification methods, carboxylation is a common and crucial technique [16,17,18]. This method offers significant advantages, including the absence of chain degradation, functionalization potential, and scalability [19]. Additionally, carboxylation is generally easier to process and handle due to the less hazardous nature of the reagents and the milder reaction conditions [20]. The carboxyl groups are versatile reactive sites that can be readily converted into other functional groups, enabling extensive polymer customization for specific applications. Vainrot et al. reported a notable example, synthesizing a polysulfone membrane through crosslinking the acid groups of the carboxylated polysulfone and an alkylated diol [21].

Despite their advantages, carboxylated polysulfone membranes still face limitations in practical water treatment applications. One major challenge is their poor rejection of multivalent ions. Typically, pressure-driven membrane processes operate under neutral pH, where the carboxylic acid groups on the membrane surface are deprotonated, forming carboxylate anions stabilized by resonance. Zeta potential experiments by Dirk et al. demonstrated that carboxylated polysulfone membranes with degrees of substitution ranging from 0.26 to 1.74 exhibited negatively charged surfaces across a pH range from 3 to 10 due to this deprotonation [22,23]. Negative charges on the carboxylated polysulfone membranes impose limitations in water treatment applications, particularly in water environments containing high-valence ions, such as hard water. In such conditions, Donnan exclusion facilitates the transport of ions via electrostatic interactions [24,25]. For instance, given Ca^2+^’s higher charge density (52 C·mm^−3^) compared to the monovalent ions Na^+^ (8 C·mm^−3^) and K^+^ (11 C·mm^−3^), Ca^2+^ is postulated to exhibit a greater affinity for the membrane, resulting in lower rejection rates [26]. Zhu et al. reported an illustrative example of the limitation of negatively charged membranes. They prepared nanocomposite polyethersulfone membranes by incorporating chitosan–montmorillonite (CS-MMT) nanosheets. These membranes exhibited a negatively charged surface and low rejection rates for divalent salts [27]. The limitations underscore the need for post-functionalization of carboxylated polysulfone membranes. However, few studies have systematically explored molecular-level post-modification strategies. Therefore, there is a clear need to further modify negatively charged PSF-COOH membranes to improve ion rejection and expand their applicability in water treatment.

In this contribution, four straightforward and readily processed methods were proposed to modify the dicarboxylated polymer, which led to the development of entirely new materials. These approaches include: (a) the preparation of a dicarboxylated polysulfone membrane followed by crosslinking the surface carboxylic groups with polyethylenimine or ethylenediamine (method A); (b) the preparation of a ethylenediamine-crosslinked dicarboxylated polysulfone membrane followed by partial hydrolysis, inducing the formation of tailored voids, while the remaining cross-linkage motifs act as brush attachments with terminal amine groups (method B); (c) the preparation of dicarboxylated polysulfone membranes with surface attached alkyl brushes (method C); (d) the formation of quaternary ammonium-functionalized dicarboxylated polysulfone membranes for antibacterial applications (method D). These strategies provide precise control over the membrane structure and functionality through molecular engineering, expanding their potential in water treatment applications. This work establishes a framework for researchers to further tailor carboxylated polysulfone and related polymers for specific performance enhancements by presenting a set of versatile modification strategies.

## 2. Experimental

### 2.1. Chemicals

Tetrahydrofuran (THF) (95%) anhydrous was purchased from Sigma-Aldrich (St. Louis, MO, USA) and distilled under nitrogen using Na/K alloy. Polysulfone (Mw = 60,000 g/mol), n-butyllithium (1.6 M) in hexane, carbon dioxide (CO_2_), branched polyethylenimine (Mw = 25,000 g/mol), ethylenediamine (EDA), 1-ethyl-3-(3-dimethylaminopropyl) carbodiimide (EDC), ethylamine, trifluoroacetic acid (TFA), and iodomethane (MeI) were received from Sigma Aldrich and used as received. N-Hydroxysuccinimide (NHS), 2-morpholonoethanesulfonic acid (MES), and hexylamine were purchased from Aaron Chemicals (San Diego, CA, USA) and used as received. Dodecylamine was purchased from Thermo Scientific (Waltham, MA, USA) and used as received. Dimethyl sulfoxide (DMSO) was obtained from Biolab (Jerusalem, Israel) and used as received.

### 2.2. Membrane Preparation

#### 2.2.1. Preparation of a Dicarboxylated Polysulfone (PSF-COOH)

Carboxylated polysulfones (PSF-COOH) with a degree of substitution of 2 were prepared using the method described by Guiver et al. [20]. This degree was selected to ensure full functionalization of the polymer backbone for subsequent post-modifications. The DS was further confirmed by back titration and NMR analysis, which showed consistent attachment of two carboxylic acid groups per repeat unit.

**Figure 1:**
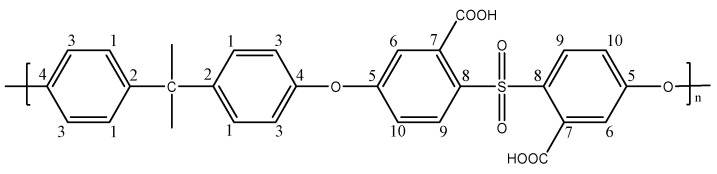
Molecular structure of PSF-COOH.

A solution of a Udel polysulfone (10.00 g, 0.0226 mol) was dissolved in THF (180 mL) and cooled to −78 °C. n-Butyllithium (1.6 M, 30 mL) was added dropwise at about one drop per second using an addition funnel under nitrogen. The solution initially exhibited a green color, later changing to a red brown solution. After the addition, the solution was stirred for 1 h, and then the cooling bath was removed. 500 g of dry ice was added to the lithiated polysulfone and vigorously mixed with a large spatula. Throughout the dry-ice addition, the flask was continuously swept with a fast stream of nitrogen to prevent moisture condensation. The resulting white polymer was left overnight to warm to room temperature, precipitated into isopropanol, and dried at 60 °C in a vacuum oven for 24 h. The dried polymer was stirred for 24 h at room temperature with a dilute aqueous HCl solution (10 wt.%) to obtain the dicarboxylic acid form. Finally, the polymer PSF-COOH, as shown in Figure 1, was washed with distilled water until reaching a pH of 7 and dried at 60 °C in a vacuum oven for 48 h and a yield of 7.3 g (83%) was obtained.

^1^H NMR (400 MHz, DMSO) δ: 1.66 (6H, s, CH_3_), 7.07 (4H, d, J = 7.58 Hz, H_3_), 7.10 (2H, s, H_10_), 7.17 (2H, d, J = 9.05 Hz, H_6_), 7.32 (4H, d, J = 7.83 Hz, H_1_), 8.04 (2H, d, J = 9.05 Hz, H_9_), 13.04 (br, COOH).

^13^C NMR (400 MHz, DMSO) δ: 30.58 (Me), 42.10 (C), 116.64 (C_10_), 117.91 (C_6_), 119.89 (C_3_), 127.53 (C_1_), 132.23 (C_7_), 133.26 (C_9_), 136.70 (C_8_), 147.18 (C_2_), 152.15 (C_4_), 161.11 (C_5_), 167.55 (C=O).

IR (KBr): 1714 cm^−1^ (C=O str), 1274 cm^−1^ (C-O str), 2900–3500 cm^−1^ (O-H str).

#### 2.2.2. Method A: Dicarboxylated Membranes Crosslinked with Polyethyleneimine or Ethylenediamine

##### Polyethylenimine (PEI) Crosslinked Dicarboxylated Polysulfones

PSF-COOH (1.00 g, 0.0019 mol) was dissolved in DMF (5.00 g, 20% *w*/*w* solution) with vigorous stirring to form a homogeneous polymer solution. The polymer was cast into a film with a stainless-steel knife with a gap of 180 µm. This film was dried in an oven at 140 °C for 3 min and left to cool at room temperature for 1 min. Then, it was immersed in a water bath to facilitate the phase inversion process and subsequently placed in a solution of 65 mM EDC and 65 mM NHS in 50 mM MES buffer with a pH of 5.5 (500 mL). After 6 h, the membrane was removed and rinsed with distilled water multiple times.

**Scheme 1:**
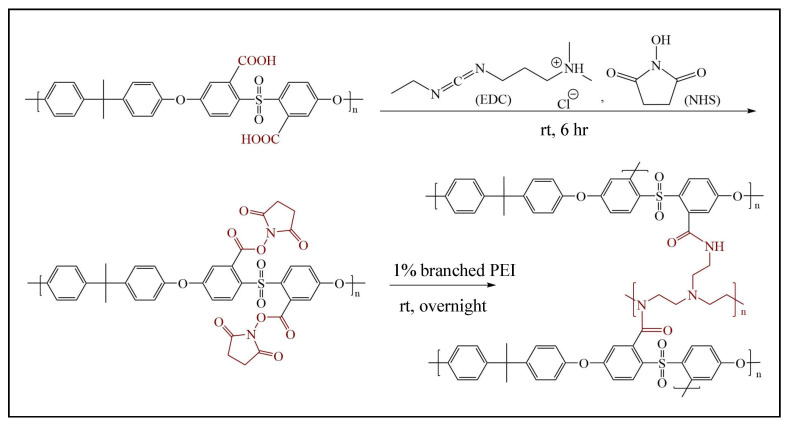
Preparation of polyethylenimine crosslinked dicarboxylated polysulfone membrane (PSF-PEI).

The activated membrane was brought into contact with a 1 wt.% branched polyethylenimine (PEI) in a water solution (500 mL) overnight to carry out the in situ crosslinking reaction between the activated PSF-COOH and the PEI molecules. The obtained membrane was rinsed with distilled water several times and stored in a distilled water bath before testing. The preparation route is illustrated in Scheme 1.

##### Ethylenediamine (EDA) Crosslinked Dicarboxylated Polysulfone

**Scheme 2:**
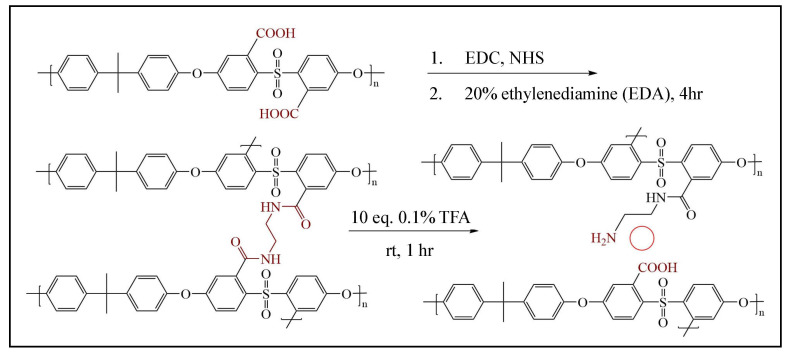
Preparation of ethylenediamine crosslinked dicarboxylated polysulfone (PSF-EDA) and partial hydrolysis of a crosslinking unit in PSF-EDA to form designed pores with aminated brushes (PSF-NH_2_).

PSF-COOH (1.00 g, 0.0019 mol) was dissolved in DMF (5.00 g, 20% *w*/*w* solution) with vigorous stirring to form a homogeneous polymer solution. EDC (0.75 g, 0.0039 mol) and NHS (0.45 g, 0.0039 mol) were added to the polymer solution and stirred for another 6 h until a yellowish and homogeneous solution was achieved. The polymer solution was left to rest for 12 h for deaeration and aging. Then, the polymer solution composed of the activated PSF-COOH was cast with a stainless-steel knife with a gap of 240 μm. The membrane was then dried in an oven at 140 °C for 3 min and cooled at room temperature for 1 min. After evaporation, the membrane was immersed in a 20 wt.% ethylenediamine solution (500 g) in water for 4 h while carrying out the in situ crosslinking reaction between the activated PSF-COOH and the EDA molecules. Finally, the membrane PSF-EDA was removed from the coagulation bath, rinsed with deionized water, and stored in deionized water before testing. The preparation route is illustrated in Scheme 2.

#### 2.2.3. Method B: Partial Hydrolysis of Crosslinking Units in PSF-EDA Membranes, Forming Designed Pores and Brushes with Terminal Amine Groups

The membrane PSF-EDA (1.00 g, 0.0015 mol) was placed in 0.1% TFA (1.14 g) in water for 1 h at room temperature. The obtained PSF-NH_2_ membranes were removed from the coagulation bath, rinsed with deionized water, and stored in deionized water before testing. The preparation route is illustrated in Scheme 2.

#### 2.2.4. Method C: Synthesis of Dicarboxylated Polysulfone Membranes Containing Various Aliphatic Brushes

##### Preparation of Dicarboxylated Polysulfone Membranes Containing Ethyl or Hexyl Brushes (PSF-C2/PSF-C6)

**Scheme 3:**
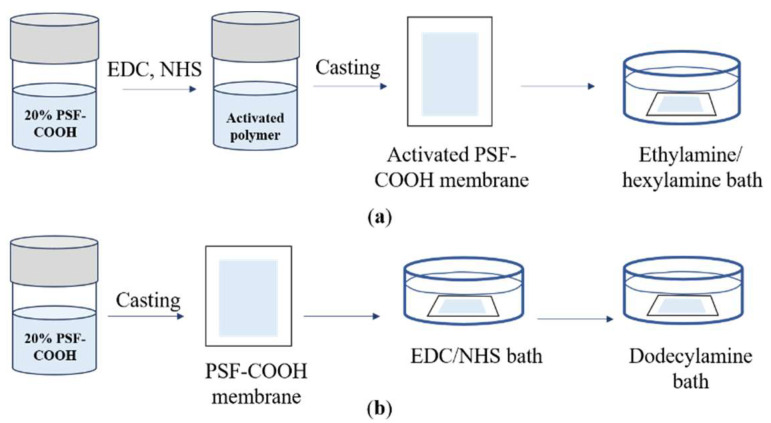
(**a**) Preparation route to PSF-C2 or PSF-C6, (**b**) preparation route to PSF-C12.

**Figure 2:**
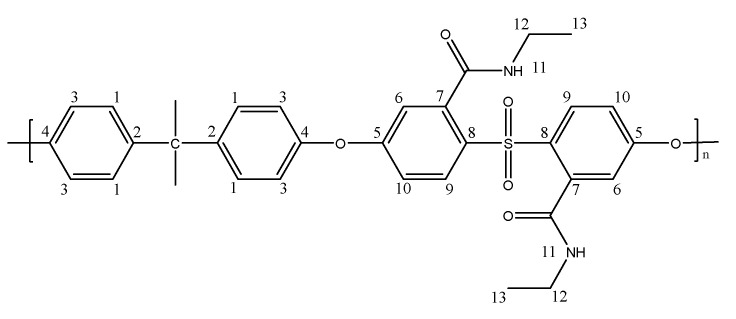
Molecular structure of PSF-C2.

**Figure 3:**
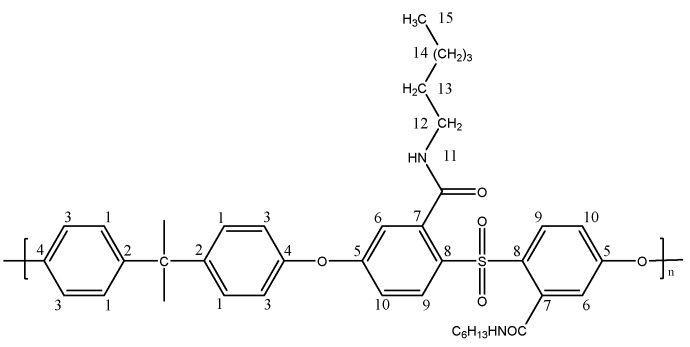
Molecular structure of PSF-C6.

PSF-COOH (1.00 g, 0.0019 mol) was dissolved in DMF (5.00 g, 20% *w*/*w* solution) at room temperature. EDC (0.75 g, 0.0039 mol) and NHS (0.45 g, 0.0039 mol) were added to the polymer solution, then stirred for 6 h until a yellowish and homogeneous solution was achieved. Then, the polymer solution composed of activated PSF-COOH was cast with a stainless-steel knife with a gap of 180 μm, which was then dried in an oven at 140 °C for 3 min and cooled at room temperature for 1 min. This film was immersed overnight in a 10% ethylamine or a 10% hexylamine solution (500 mL) to induce the amide formation and the phase inversion process. Finally, the obtained membranes, PSF-C2 (1.00 g, 90% yield) and PSF-C6 (1.13 g, 86% yield), as shown in Figure 2 and Figure 3, were taken out from the coagulation bath, rinsed with distilled water to remove any unreacted amine, and stored in a distilled water bath before testing. The preparation route is illustrated in Scheme 3a. In both cases, all the carboxylic acid moieties were reacted as confirmed by NMR.

^1^H NMR (400 MHz, DMSO) δ: 1.05 (6H, m, H_1_), 1.64 (6H, s, H_2_), 3.18 (4H, m, H_3_), 6.84 (2H, s, H_4_), 7.01 (6H, m, H_5_, H_3_), 7.30 (4H, d, J = 7.70 Hz, H_6_), 8.12 (2H, d, J = 8.80 Hz, H_7_), 8.39 (2H, s, H_8_).

^13^C NMR (400 MHz, DMSO) δ: 14.27 (C_13_), 30.61 (Me), 34.15 (C_11_), 42.10 (C), 116.60 (C_10_), 117.00 (C_6_), 119.76 (C_3_), 128.61 (C_1_), 132.14 (C_7_), 133.88 (C_9_), 140.07 (C_8_), 147.07 (C_2_), 152.27 (C_4_), 160.92 (C_5_), 166.61 (C=O).

IR(KBr): 1649 (C=O str, amide group).

^1^H NMR (400 MHz, DMSO) δ: 0.81 (6H, m, H_15_), 1.23 (12H, m, H_14_), 1.46 (4H, t, J = 6.17 Hz, H_13_), 1.65 (6H, s, Me), 3.13 (4H, m, H_12_), 6.83 (2H, s, H_6_), 7.04 (6H, m, H_3_, H_10_), 7.30 (4H, s, H_1_), 8.13 (2H, d, J = 9.05 Hz, H_9_), 8.42 (2H, s, H_11_).

^13^C NMR (400 MHz, DMSO) δ: 13.93 (C_15_), 22.09 (C_14_), 26.04 (C_14_), 27.80 (C_14_), 28.53 (C_13_), 30.55 (Me), 30.98 (C_12_), 42.10 (C), 116.38 (C_10_), 116.88 (C_6_), 119.67 (C_3_), 128.48 (C_1_), 132.10 (C_7_), 133.82 (C_9_), 140.06 (C_8_), 146.67 (C_2_), 152.15 (C_4_), 160.77 (C_5_), 166.58 (C=O).

IR(KBr): 1649 (C=O str, amide group).

##### Preparation of a Dicarboxylated Polysulfone Membrane Containing Dodecylamine Brushes (PSF-C12)

PSF-COOH (1.00 g, 0.0019 mol) was dissolved in DMF (5.00 g, 20% *w*/*w* solution) at room temperature and cast into a film of 180 µm thickness using a stainless-steel blade knife. The membrane was then dried in an oven at 140 °C for 3 min and cooled at room temperature for 1 min. This film was immersed in a water bath to facilitate the phase inversion process and subsequently placed in a solution of 65 mM EDC and 65 mM NHS in 50 mM MES buffer (500 mL) with a pH of 5.5. After 6 h, the membrane was removed and rinsed multiple times with distilled water.

**Figure 4:**
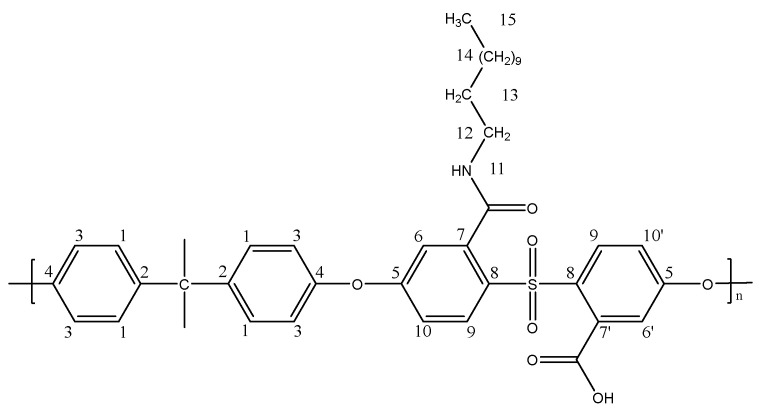
Molecular structure of PSF-C12.

The activated membrane was brought in contact with a solution saturated with dodecylamine overnight, followed by washing with distilled water to remove unreacted amine. The obtained membrane, PSF-C12 (1.11 g, 85% yield), as shown in Figure 4, was stored in a distilled water bath before undergoing testing. NMR and FTIR analyses confirmed that approximately 50% of the carboxylic acid groups were replaced. The preparation route is illustrated in Scheme 3b.

^1^H NMR (400 MHz, DMSO) δ: 0.81 (3H, t, J = 6.69 Hz, H_15_), 1.19 (18H, m, H_14_), 1.40 (2H, m, H_13_), 1.65 (6H, m, Me), 3.29 (2H, m, H_12_), 6.83 (2H, m, H_6_, H_6′_), 7.04 (6H, m, H_3_, H_10_, H_10′_), 7.80 (6H, m, H_1_, H_9_), 8.86 (1H, m, H_11_), 10.51 (br, COOH).

^13^C NMR (400 MHz, DMSO) δ: 13.93 (C_15_), 22.10 (C_13_), 25.88 (C_14_), 27.02 (C_14_), 28.56 (C_14_), 28.72 (C_14_), 28.86 (C_14_), 28.96 (C_14_), 29.03 (C_14_), 29.06 (C_14_), 30.98 (Me), 31.30 (C_13_), 38.54 (C_12_), 42.10 (C), 114.88 (C_7_), 116.16 (C_10_), 116.78 (C_3_), 117.80 (C_10′_), 119.77 (C_7_), 122.26 (C_6_), 128.41 (C_6′_), 128.88 (C_9_), 129.33 (C_1_), 129.79 (C_7′_), 130.38 (C_8_), 146.76 (C_2_), 150.09 (C_4_), 162.38 (C_5_), 167.34 (C=O).

IR(KBr): 1701 (C=O str, carboxylic acid).

#### 2.2.5. Method D: Formation of Quaternary Ammonium Motifs at the Surface of the Crosslinked PSF-PEI or PSF-NH_2_ Membranes

**Scheme 4:**
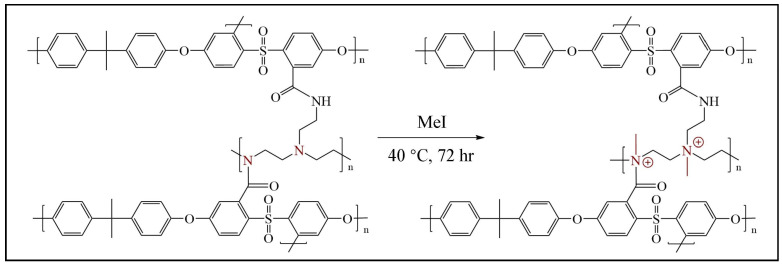
Preparation of quaternary ammonium polyethylenimine crosslinked carboxylated polysulfone membrane (QA-PEI-PSF). I^−1^ ions are not shown for clarity.

The PSF-PEI (1.00 g, 0.0016 mol) or PSF-NH_2_ (1.00 g, 0.0016 mol) membranes were immersed in an excess of MeI (4.00 mL) for 72 h at 42 °C under reflux conditions, with the setup covered by aluminum paper to prevent exposure to sunlight. The color of the membrane changed to yellowish after 48 h. The obtained membranes, QA-PSF-PEI and QA-PSF-NH2, were rinsed with distilled water and stored in a water bath before undergoing permeance measurement (Scheme 4).

### 2.3. Membrane Characterizations

The surface chemical composition of the membranes was characterized by Attenuated Total Reflectance–Fourier Transform Infrared Spectroscopy (ATR-FTIR, Bruker, Tensor 27, Billerica, MA, USA). To further verify the presence of functional groups in crosslinked polymers, the D_2_O exchange technique was employed in FTIR, which involves observing changes in vibrational bands associated with exchangeable protons. When the polymer was reacted with deuterium oxide (D_2_O), the exchangeable protons (H) in the sample underwent isotopic exchange with the deuterium atoms (D) from D_2_O. This process, known as hydrogen–deuterium exchange (H/D exchange), follows the relation:

(1) $$\frac{\nu_{D}}{\upsilon_{H}}=\sqrt{\frac{\left(\frac{m_{D}+m_{1}}{m_{D}m_{1}}\right)}{\left(\frac{m_{H}+m_{1}}{m_{H}m_{1}}\right)}}=\sqrt{\frac{m_{H}}{m_{D}}}=0.707$$

where *υ* is the frequency in cm^−1^, m is the atom’s mass.

For samples containing carboxylic acid or amine groups, dilution with D_2_O results in the original O-H or N-H stretching vibration bands decreasing in intensity or disappearing. Concurrently, new bands corresponding to O-D or N-D stretching vibrations appear at lower frequencies, typically in the range of 2400–2700 cm^−1^, according to Equation (1). This shift in vibrational bands confirms the presence of carboxylic acid and amine groups in the polymer structure, validating the functionalization process and the successful synthesis of the desired crosslinked polymers.

The surface and cross-sectional morphologies of the membrane were characterized using Field Emission Scanning Electron Microscopy (TESCAN Vega-II, Brno, Czech Republic). The membrane for cross-section morphology observation was scraped off from the supporting fabric and cryogenically broken in liquid nitrogen to hunt for a distinct fracturing frame. Membrane samples were gold-suspended before SEM observation.

Thermal properties were determined using a Differential Scanning Calorimetry (DSC) Q10 V9.0 Build 275 calorimeter. DSC measurements were performed from 25 to 300 °C with a scanning rate of 10 °C min^−1^ in an N_2_ atmosphere for three cycles.

### 2.4. Filtration Experiments

Filtration testing of membranes was performed on a lab-scale dead-end filtration system. Active membranes with an area of 0.0021 m^2^ were located in a testing cell and pre-compacted for 1 h at 10 bars before the measurement. Additionally, all membranes were self-supporting. All filtration experiments in this study were conducted under constant conditions (20 °C, neutral pH) to ensure comparability across membranes. Although variations in pH and temperature may affect salt rejection behavior, these effects were not systematically investigated here and remain a subject for future research.

The filtration permeance of deionized water and salt solution was measured from 2 to 10 bar and calculated using Equation (2).

(2) $$J_{V}=\frac{Q}{A}$$

Parameters Q and A are the volumetric flow rate and the membrane active area.

Rejection experiments were conducted at 2 bars with identical feed solutions of 0.1 wt.% CaCl_2_, NaCl, and Na_2_SO_4_, and pre-filtrated for 1 h before measuring. The permeate samples in rejection were collected only after the complete water removal and repeated cell washings with the feed solutions. Salt rejection calculation was based on conductivity (Equation (3)), which shows the calculation of salt rejection according to conductivity measurements of the feed and permeate. A conductometer (PP1 MironL—TechPro2, The Myron L, Carlsbad, CA, USA) was used to measure the conductivity of both permeate and feed solutions.

(3) $$R=1-\frac{C_{P}}{C_{F}}$$

C_F_ and C_P_ are the conductivities of the feed solution and permeate, respectively.

### 2.5. Bacterial Attachment and Biofilm Formation

A swab inoculation assay was employed to investigate the initial bacterial deposition and biofilm formation on the quaternary ammonium membranes. The bacterial inoculum was serially diluted in phosphate-buffered saline (PBS) supplemented with 5% glycerol to achieve a final concentration of 6 × 10^4^ colony-forming units (CFUs) per milliliter. A 50 µL bacterial suspension was pipetted onto the control parafilm, a pristine polysulfone, and the quaternary ammonium membranes (dimensions 1.0 × 1.0 cm) were spread homogenously by rolling a cotton swab over the surface. The membranes were then allowed to contact the bacterial suspension under ambient conditions for 0 and 90 min. After the designated periods, the membranes were sampled using a contact plate by pressing the membrane surface for 10 s. The contact plates were then incubated for 24 h at 35 °C to allow for bacterial growth. Finally, the number of CFUs was manually counted to determine the antibacterial efficacy of the membranes [28,29].

## 3. Results and Discussion

### 3.1. Method A: Dicarboxylated Membranes Crosslinked with Polyethyleneimine or Ethylenediamine

Branched polyethylenimine (PEI) is a hyperbranched, water-soluble, and hydrophilic polymer known for its non-toxic nature, making it widely applicable in various fields. This study introduces PEI as a novel crosslinker for the dicarboxylated polysulfone (PSF-COOH), leveraging its high density of amine groups to enhance membrane properties. In comparison, ethylenediamine (EDA), a short-chain molecule, is generally used when more controlled, lower complex crosslinking is desired. This study investigates PEI and EDA as crosslinkers for PSF-COOH to evaluate the differences in crosslinking behavior, membrane morphology, and overall performance.

Notably, the PSF-PEI membrane was post-modified directly on the PSF-COOH membrane, whereas the PSF-EDA membrane was post-modified on a PSF-COOH membrane pretreated with EDC and NHS (activated PSF-COOH membrane). The casting thickness for the PSF-PEI membrane was 180 µm, while for the PSF-EDA membrane, it was 240 µm. The PSF-EDA membrane could not be synthesized at 180 µm due to excessive swelling of the activated PSF-COOH membrane, necessitating a thicker membrane for successful fabrication.

**Figure 5:**
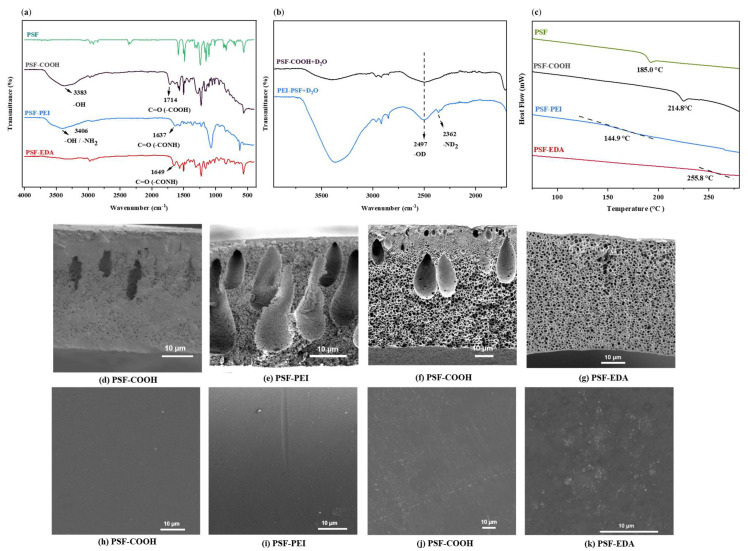
(**a**) ATR-FTIR spectra of PSF-COOH, PSF-PEI, and PSF-EDA membranes; (**b**) ATR-FTIR spectra of PSF-COOH and PSF-PEI membranes after H/D exchange with D_2_O; (**c**) DSC results of membranes, noting that Tg of branched PEI is −52 °C [31]. Cross-sectional SEM images of membranes: (**d**) PSF-COOH with a casting thickness of 180 µm, measured thickness of 34.4 µm; (**e**) PSF-PEI, thickness 30.2 µm; (**f**) PSF-COOH with a casting thickness of 240 µm, measured thickness of 47.5 µm; (**g**) PSF-EDA, thickness 47.3 µm. Surface SEM images of membranes: (**h**) PSF-COOH with a casting thickness of 180 µm; (**i**) PSF-PEI; (**j**) PSF-COOH with a casting thickness of 240 µm; (**k**) PSF-EDA.

Figure 5a presents the complete ATR-FTIR spectra of PSF-COOH, PSF-PEI, and PSF-EDA membranes. In the PSF-COOH membrane, the peak at 1714 cm^−1^ confirms the presence of carboxylic acid groups. The appearance of peaks at 1637 cm^−1^ and 1649 cm^−1^ in the PSF-PEI and PSF-EDA membranes, respectively, corresponds to the stretching mode of newly formed amide bonds, indicating successful crosslinking. The difference in the amide peak characteristics between PSF-PEI and PSF-EDA can be attributed to the distinct structures of the crosslinkers. PEI, being a hyperbranched polymer with a distribution of primary, secondary, and tertiary amines, results in a broader and more complex amide region. In contrast, EDA contains only primary amines, leading to sharper and better-defined amide peaks in the FTIR spectra. In addition, the PSF-EDA membrane lacks the broad peak around 3390 cm^−1^, suggesting the replacement of hydroxyl (OH) groups upon crosslinking.

Figure 5b displays the ATR-FTIR spectra of PSF-COOH and PSF-PEI membranes after hydrogen–deuterium (H/D) exchange with D_2_O. In the PSF-COOH membrane, the broad peak at 2497 cm^−1^ confirms the presence of OD groups, originating from the carboxylic acid. For the PSF-PEI membrane, the peak at 2362 cm^−1^ indicates the formation of ND_2_ groups, further verifying the crosslinking of polyethylenimine.

According to the Differential Scanning Calorimetry (DSC) results (Figure 5c), the glass transition temperature (Tg) of PSF-PEI decreased compared to PSF-COOH. This reduction is attributed to the flexible, aliphatic structure of PEI. When introduced into the polymer network, PEI may act as a soft segment, increasing chain mobility rather than restricting it and ultimately lower the Tg of crosslinking material.

In contrast, the Tg of PSF-EDA increased sharply, indicating stronger intermolecular forces within the polymer matrix due to more effective crosslinking. The smaller, less sterically hindered EDA molecules facilitate closer chain interactions, reinforcing the polymer network and raising Tg [30].

The cross-sectional SEM images (Figure 5) reveal that the PSF-COOH and PSF-PEI membranes exhibit similar finger-like morphologies with comparable thicknesses, as do the PSF-PEI membranes synthesized through the post-modification of PSF-COOH membranes.

In contrast, the SEM images of the PSF-EDA membrane show distinct morphologies and thicknesses compared to the PSF-COOH, characterized by a sponge-like structure. The PSF-EDA was post-modified on the activated PSF-COOH, meaning its morphology was influenced by the precipitation rate of the activated carboxylated polymer and the exchange rate between the solvent (DMF) and the non-solvent (water). The high concentration of EDA (20%) increased the viscosity of the coagulation bath, reducing the solvent exchange rate and ultimately resulting in a sponge-like structure.

The surface SEM images (Figure 5) demonstrate minimal defects across all membrane surfaces, indicating that the salt separation mechanism is not primarily driven by size exclusion. The absence of large surface pores suggests that the membranes may rely on alternative separation mechanisms, such as Donnan exclusion or dielectric exclusion, to achieve effective salt rejection. Although SEM was used to observe membrane morphology, it is not suitable for precise pore size quantification, especially for internal pores. Techniques such as nitrogen adsorption (BET) or porometry could provide more accurate data. Due to experimental constraints, these were not included in the current study.

**Figure 6:**
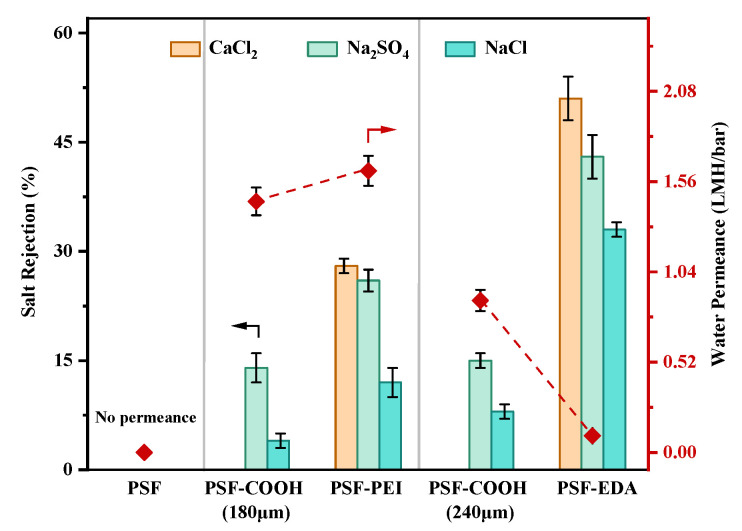
Plots of permeance of distilled water and salt rejection of pristine PSF, PSF-COOH, PSF-PEI and PSF-EDA. Salt permeance data are not presented, as they differ by less than 2% from water permeance.

Figure 6 compares the permeance and salt rejection of various membranes, including the pristine PSF, PSF-COOH, PSF-PEI, and PSF-EDA. The pristine polysulfone exhibited no measurable permeance under 10 bar. The PSF-COOH membrane, containing two carboxylic acid groups, demonstrated a higher permeance. The water permeance of PSF-PEI showed a slight increase as compared to the PSF-COOH, primarily due to the inherent hydrophilicity of the PEI moiety [32]. Additionally, the permeance of PSF-EDA decreased significantly, reaching only one-tenth of that of PSF-COOH, suggesting the formation of a denser crosslinked network that restricts water transport.

In salt rejection experiments, the PSF-PEI and PSF-EDA membranes achieved significantly higher salt rejection than the PSF-COOH membrane. Due to the Donnan exclusion, the PSF-COOH membrane exhibited no rejection of CaCl_2_, demonstrating its inability to filter out positive divalent ions [33]. However, crosslinking effectively reduced charge distribution, demonstrating its positive impact on ion rejection.

The analysis of the PSF-PEI and PSF-EDA membranes found that their salt rejection followed the order of NaCl < Na_2_SO_4_ ≈ CaCl_2_. Considering that the membrane polymers remain electrically neutral under neutral water conditions, dielectric exclusion likely plays a dominant role in the solute separations. The comparable rejection of calcium (Ca^2+^) and sulfate (SO_4_^2−^) ions, which is more effective than that of monovalent ions (Na^+^), can be attributed to the higher energy cost required for these multivalent ions to shed their solvation shells. This suggests that the membrane’s separation efficiency is driven by the differential solvation energy of ions, favoring the exclusion of multivalent species over monovalent ions [25].

### 3.2. Method B: Hydrolysis of Crosslinking Units in PSF-EDA Membranes, Forming Designed Pores and Brushes with Terminal Amine Groups

As shown above, utilizing method A, ethylenediamine limits the membrane’s permeance. To address this, partial hydrolysis of certain cross-linkages was performed to create voids within the crosslinked layer, increasing the membrane porosity [21]. This approach balances permeability while maintaining its mechanical strength and chemical resistance, preventing excessive swelling or shrinkage. Moreover, the remaining crosslinking motifs, which are still attached to the membrane from one side, function as brush-like attachments on the membrane. These motifs prevent the polymer from reverting to its original carboxylated polysulfone state, influencing the adequate pore size and enhancing filtration performance.

**Figure 7:**
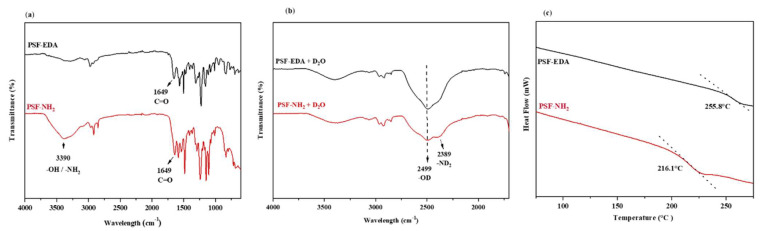
(**a**) ATR-FTIR spectra of membranes PSF-EDA and PSF-NH_2_; (**b**) ATR-FTIR spectra of membranes PSF-EDA, PSF-NH_2_ after H/D exchange with D_2_O; (**c**) DSC results of both membranes.

Figure 7a presents the complete ATR-FTIR spectra of PSF-EDA and PSF-NH_2_ membranes. The peak at 1649 cm^−1^ in the PSF-EDA and PSF-NH_2_ suggests the presence of the C=O bond. The PSF-NH_2_ membranes exhibit a broad signal at 3390 cm^−1^, attributed to the hydroxyl and amine groups.

To further confirm the presence of the primary amine group in PSF-NH_2_, hydrogen–deuterium (H/D) exchange with D_2_O was applied to compare the PSF-EDA and the PSF-NH_2_ membranes (Figure 7b). For the PSF-EDA membrane, the broad peak at 2499 cm^−1^ indicates the presence of OD groups from residual carboxylic acid, while the PSF-NH_2_ membrane shows an increased peak at 2389 cm^−1^, attributed to the ND_2_ groups.

According to the DSC results (Figure 7c), the glass transition temperature (T_g_) of PSF-NH_2_ decreased compared to PSF-EDA, due to the reduced crosslink density.

**Figure 8:**
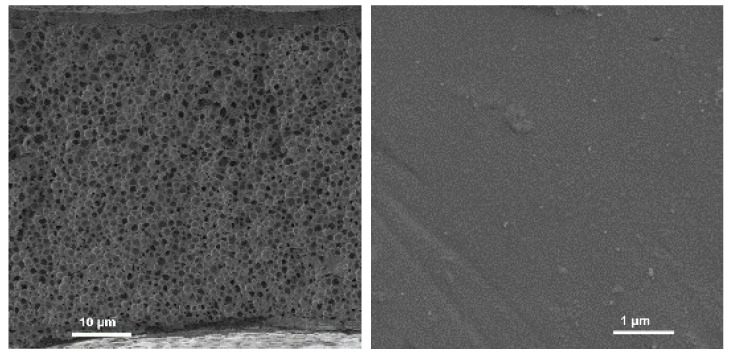
Cross-sectional and surface SEM images of membranes PSF-NH_2_, thickness 55.7 µm.

The SEM images of PSF-NH_2_ (Figure 8) reveal similar morphologies to PSF-EDA, characterized by a sponge-like structure. The relatively consistent membrane thickness suggests that swelling from hydrolysis was effectively mitigated. Additionally, the surface exhibits minimal defects after acid hydrolysis, indicating that the separation mechanism is likely governed primarily by dielectric exclusion. While the extent of hydrolysis in Method B was not quantified, FTIR, DSC, and SEM results collectively indicate that partial hydrolysis was successfully achieved.

**Figure 9:**
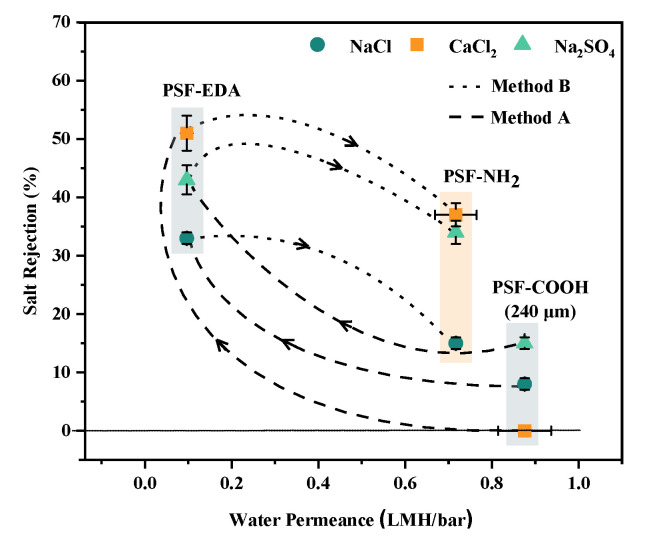
Plots of permeance of distilled water and salt rejection of PSF-COOH, PSF-EDA and PSF-NH_2_. Salt permeance data are not presented, as they differ by less than 2% from water permeance.

Figure 9 compares the membrane performance using method A and method B, illustrating the trends in water permeance and salt rejection for PSF-COOH, PSF-EDA, and PSF-NH_2_. As observed in method A, the water permeance of PSF-EDA was significantly lower than that of PSF-COOH, while its salt rejection was markedly higher. Upon partial hydrolysis of the crosslinkers, the PSF-NH_2_ membrane achieved a remarkable sevenfold increase in permeance. Notably, this hydrolysis process did not significantly reduce salt rejection, with the PSF-NH_2_ membrane achieving 37% rejection for CaCl_2_ and 34% for Na_2_SO_4_. This increase in permeance without substantial loss of salt rejection efficiency suggests that controlled hydrolysis effectively enhances membrane permeability while maintaining selective separation.

Since PSF-NH_2_ is neutral, dielectric exclusion is the dominant separation mechanism. The observed rejection trend follows the order CaCl_2_ ≈ Na_2_SO_4_ > NaCl, indicating a preference for excluding multivalent ions over monovalent ones.

### 3.3. Method C: Synthesis of Dicarboxylated Polysulfone Membranes Containing Various Aliphatic Brushes

Method C investigated the effect of short-chain brushes (PSF-NH_2_) with a two-carbon length. To further explore the impact of brush lengths on the membrane performance, novel membranes incorporating aliphatic chains of varying lengths were developed. By integrating hydrophobic brushes, a series of modified polysulfone membranes, denoted PSF-(CONHC_2_H_5_)_2_, PSF-(CONHC_6_H_13_)_2_, and PSF-CONHC_12_H_25_ (abbreviated as PSF-C2, PSF-C6, and PSF-C12, respectively) were synthesized and thoroughly characterized using NMR and FTIR. These materials introduce tunable hydrophobicity and structural variation, allowing a systematic evaluation of brush length effects on membrane properties and performance.

**Figure 10:**
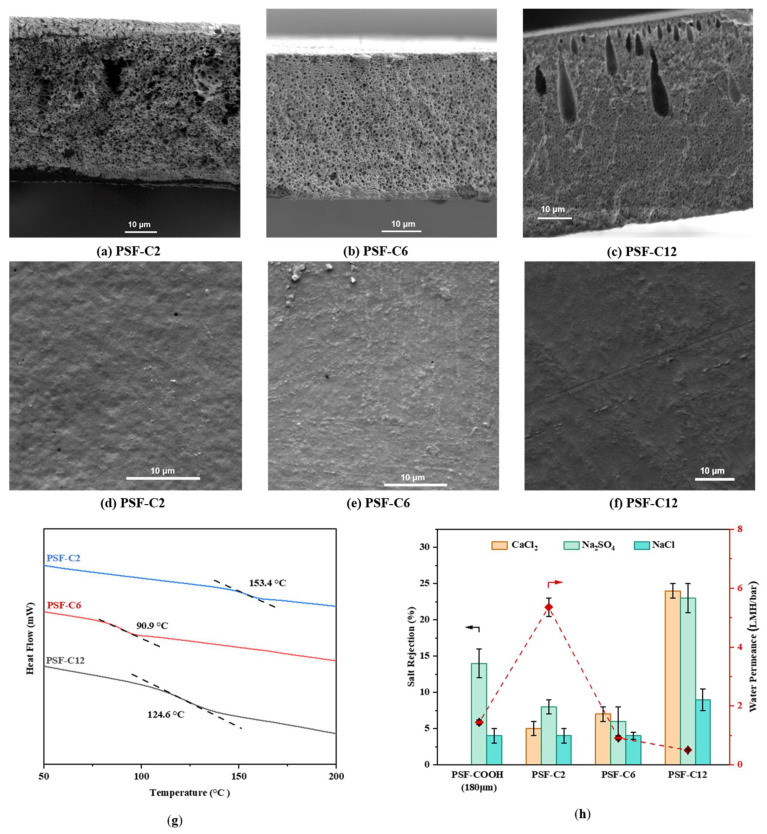
Cross-sectional SEM images of membranes. (**a**) PSF-C2, thickness 51.3 µm; (**b**) PSF-C6, thickness 32.6 µm; (**c**) PSF-C12, thickness 47.0 µm. Surface SEM images of membrane (**d**) PSF-C2; (**e**) PSF-C6; (**f**) PSF-C12; (**g**) DSC results of PSF-C2, PSF-C6 and PSF-C12; (**h**) plots of permeance of distilled water and salt rejections. Salt permeance data are not presented, as they differ by less than 2% from water permeance.

The cross-sectional SEM images (Figure 10) illustrate the morphologies and thicknesses of the PSF-C2, PSF-C6, and PSF-C12 membranes. The PSF-C2 membrane exhibits a sponge-like structure with large voids, while the PSF-C6 membrane displays a typical sponge-like structure. The PSF-C12 membrane, post-modified from PSF-COOH, features a finger-like structure. Among the membranes prepared, the PSF-C6 has the thinnest thickness.

The PSF-C2 and PSF-C6 membranes exhibit similar morphologies and thicknesses since they both result from the post-modification of the activated PSF-COOH membranes. However, observed differences are attributed to the phase inversion process, primarily influenced by the duration required for liquid–liquid demixing. The strong hydrophobicity of hexylamine, which is higher than that of ethylamine, leads to its incomplete dissolution in water at high concentrations (10%), resulting in aggregation or micelle formation. This aggregation increased the viscosity of the solution and hindered the influx of nonsolvent (water) during the immersion precipitation process. Consequently, PSF-C6 forms denser membrane structures with lower porosity compared to PSF-C2 and has a thinner thickness.

According to the DSC results (Figure 10g), the complete replacement of the hydrogen bonds decreased the T_g_ for the membranes PSF-C2, PSF-C6, and PSF-C12. However, the NMR results show that PSF-C12 retained 50% of the carboxylic acid groups during its formation, contributing to its higher Tg compared to the other two membranes.

Figure 10h illustrates the water permeance of various membranes, including PSF-COOH, and three PSF-Cn derivatives. The water permeance of the PSF-C2 membrane exhibited a remarkable increase of 325% as compared to that of PSF-COOH.

In contrast, the permeances of the PSF-C6 and PSF-C12 membranes were slightly lower than those of the PSF-COOH membrane. The decrease in permeance can be attributed to the increased hydrophobicity of the longer alkyl hydrophobic side chains, which decreases the wettability of the membrane surface and reduces water permeance. The longer alkyl side chains also contribute to a more constrained free volume within the polymer matrix, further decreasing the permeance. In addition, the PSF-C2 membrane exhibited the greatest thickness yet showed the highest water permeance, suggesting that the rate-limiting step for water transport is governed by the surface layer rather than the bulk membrane thickness.

Among the salt rejection characteristics of the membranes, the PSF-C12 membrane exhibits the best results. Its rejection for CaCl_2_ increased from 0% to 24%, while rejections for NaCl and Na_2_SO_4_ doubled. This highly selective performance is due to the enhanced dielectric exclusion mechanism of the membranes [24]. The strong hydrophobicity of PSF-C12, driven by its long alkyl chains, results in a lower dielectric constant, which increases the energy barrier for ion permeation. As a result, ions with higher charges, such as Na_2_SO_4_ and CaCl_2_, experience more substantial solvation energy constraints, leading to the highest observed rejection. In contrast, the higher dielectric constants in the membranes like PSF-C2 and PSF-C6 result in a lower salt rejection efficiency due to their shorter alkyl chains and lower hydrophobicities.

Overall, the PSF-C2 membrane exhibits a significant increase in permeance but lower salt rejection. In contrast, the PSF-C12 membrane achieves remarkably higher rejection for all salts, albeit at the cost of reduced permeance. These findings emphasize the critical role of polymer brush lengths and density in governing membrane performance. The longer polymer brushes in PSF-C12 create a more effective barrier to ion transport, enhancing selectivity while reducing permeability.

### 3.4. Method D: Formation of Quaternary Ammonium Motifs at the Surface of the Crosslinked PSF-PEI or PSF-NH_2_ Membranes

The PSF-PEI and PSF-NH_2_ membranes can be readily modified into quaternary ammonium polymers, which enhances their performance by preventing biofilm formation while maintaining high salt rejections.

The confirmation of the quaternary ammonium (QA) salt was carried out through a silver nitrate test. The QA membrane was brought in contact with a 0.1 M silver nitrate water solution. The formation of precipitated AgI was indicated by the solution becoming turbid upon exposure to sunlight, which provided evidence of the presence of the iodide salt and the formation of the quaternary ammonium salt. The reaction for this test is:

$$I^{-}+{AgNO}_{3}\to AgI\;↓+{{NO}_{3}}^{-}$$

**Figure 11:**
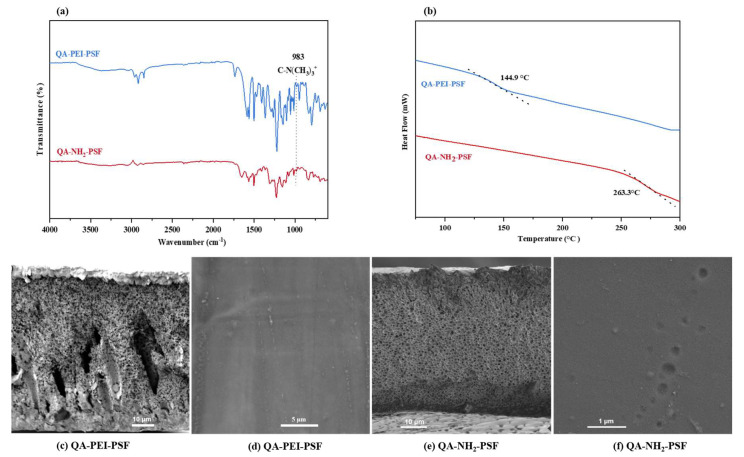
(**a**) ATR-FTIR spectra of membranes QA-PEI-PSF, QA-NH_2_-PSF; (**b**) DSC results of membranes. Cross-sectional SEM images of membranes: (**c**) QA-PEI-PSF, thickness 68.9 µm; (**d**) QA-PEI-PSF; (**e**) QA-NH_2_-PSF, thickness 52.8 µm. Surface SEM images of membrane: (**f**) QA-NH_2_-PSF.

Figure 11a presents the complete ATR-FTIR spectra of QA-PEI-PSF and QA-NH_2_-PSF membranes. For the QA-PEI-PSF membrane, the increased peak intensity at 2916 cm^−1^ and the decreased intensity around 3400 cm^−1^ suggest the complete substitution of the hydrogens in the amine groups with methyl groups. QA-PSF-NH_2_ membranes do not show the broad peak around 3390 cm^−1^, indicating the replacement of the OH groups. In addition, both spectra feature an increased signal at 983 cm^−1^, indicative of the C-N(CH_3_)_3_^+^ stretching.

Figure 11b shows that the T_g_ of the QA-PEI-PSF polymer is smaller than that of the PSF-PEI. This results from the significant reduction in hydrogen bonds from substituting the hydrogens of the primary and secondary amines with methyl groups.

The T_g_ of QA-PSF-NH_2_ was significantly higher than that of PSF-NH_2_. This is because hydrogen bonds, which are relatively weak intermolecular forces, were replaced by ionic interactions. The introduction of quaternary ammonium salts created strong electrostatic attractions between the positively charged ammonium groups and the unreacted negatively charged carboxylate ions (COO-), requiring more thermal energy to achieve the polymer mobility necessary for the transition from a glassy to a rubbery state. In addition, introducing bulky quaternary ammonium groups reduces the available free volume for polymer motions, further contributing to a higher T_g_ [34,35].

The SEM images in Figure 11 show that the QA-PEI-PSF membrane has a larger thickness due to swelling, whereas the QA-NH_2_-PSF membrane maintains a similar thickness to that of PSF-NH_2_. This is attributed to ionic interactions that counteract the swelling effect, stabilizing the membrane structure.

**Figure 12:**
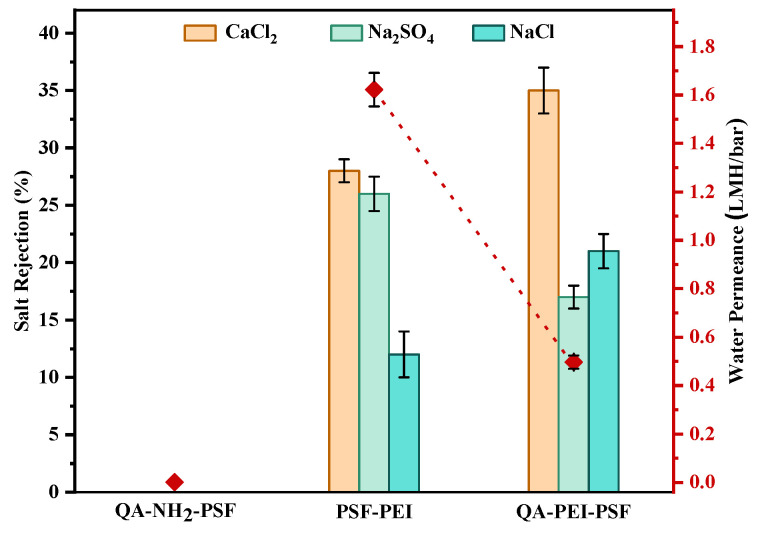
Plots of permeance of distilled water and salt rejections. Salt permeance data are not presented, as they differ by less than 2% from water permeance.

Figure 12 compares the water permeance and salt rejections of the PSF-PEI and the QA-PEI-PSF membranes. The water flux through the QA-PSF-NH_2_ membrane was too small to measure accurately at 10 bars (smaller than 0.01 L/m^2^·h). Due to electrostatic attractions and increased thickness, the permeance of the QA-PEI-PSF membrane was reduced to one-third of that of the PSF-PEI membrane. The substantial reductions in water permeance for both membranes can be primarily attributed to the increase in membrane thickness after quaternization.

In salt rejection experiments, the QA-PEI-PSF membrane exhibited the following order of rejection: CaCl_2_ > NaCl > Na_2_SO_4_. As the membrane incorporated quaternary ammonium salts and carried a positive charge, its salt separation mechanism relied mainly on the Donnan exclusion. For larger cations like Ca^2+^ the positively charged membrane exerted a stronger repulsive force, impeding their transport across the membrane and resulting in enhanced rejections. Conversely, for larger anions, SO_4_^2−^, the membrane demonstrated a lower rejection [24,33].

To evaluate the antibacterial performance, Escherichia coli (*E. coli*, a Gram-negative bacterium) and *Bacillus* species (Gram-positive bacteria) were used. The tested membranes include a control parafilm, a polysulfone (PSF), the QA-NH_2_-PSF, and the QA-PEI-PSF. Table 1 compares the bacterial growth of these membranes by quantifying the colony-forming units (CFUs) at the two time points.

The data from the table indicate significant differences in bacterial attachment and biofilm formation among the various membranes.

Bacterial adhesion was evaluated based on CFU counts at 0 min. The control parafilm and unmodified PSF membranes exhibited low bacterial attachment, consistent with their hydrophobic and electrically neutral surface properties [36].

The QA-PEI-PSF membrane exhibited a strong affinity for *E. coli*, with 257 CFUs, but had a significantly lower affinity for *Bacillus*, with only 8 CFUs. Similarly, the QA-NH_2_-PSF membrane showed high initial deposition for *E. coli* (190 CFUs) and low adhesion for *Bacillus* (4 CFUs). The higher deposition of *E. coli* compared to *Bacillus* can be attributed to the higher overall negative charge density of *E. coli* [37]. The overall bacterial affinity of the QA-PEI-PSF membrane was stronger than that of the QA-NH_2_-PSF membrane, likely due to the higher concentration of positively charged quaternary ammonium groups in the QA-PEI-PSF membrane.

The change in bacterial counts over time provides insight into biofilm growth rates on the different membrane surfaces. Over 90 min, the control parafilm exhibited a dramatic increase in *E. coli* CFUs, with a growth rate of 4900%, indicating rapid biofilm formation. Similarly, the unmodified PSF membrane showed substantial increases in bacterial growth, with a 1195% rise in *E. coli* CFUs and a 363% rise in *Bacillus* CFUs. These significant growth rates on both the parafilm and PSF membranes suggest that neither material effectively inhibits biofilm formation, highlighting their limited antibacterial properties compared to the quaternary ammonium-functionalized membranes.

In contrast to the control and unmodified PSF membranes, the quaternary ammonium-functionalized membranes demonstrated significantly reduced biofilm formation. The QA-PEI-PSF membrane exhibited a 34% reduction in *E. coli* colony-forming units (CFUs) and a complete eradication of *Bacillus*. Similarly, the QA-NH_2_-PSF membrane showed a 52% reduction in *E. coli* CFUs and a 50% reduction in *Bacillus* CFUs. The higher inhibition rate of the QA-NH_2_-PSF membrane can be attributed to its alkyl brush (ethyl amine group), which interacts more effectively with and disrupts bacterial cell membranes, enhancing its antimicrobial activity [38].

The observed difference in reduction rates between *E. coli* (Gram-negative) and *Bacillus* (Gram-positive) suggests that Gram-positive bacteria are more susceptible to the antibacterial effects of these functionalized membranes. This is likely due to the penetration efficiency of the hydrophobic alkyl chains, which are more effective in disrupting the thicker peptidoglycan layer of Gram-positive bacteria than the outer membrane of Gram-negative bacteria. This highlights the potential of these quaternary ammonium-functionalized membranes for targeted antibacterial applications, particularly against Gram-positive pathogens [39,40].

In conclusion, while the quaternary ammonium-functionalized membranes initially promoted bacterial deposition due to their positively charged surfaces, the antibacterial activity of the quaternary amines ultimately dominated, effectively preventing biofilm formation. These findings suggest the potential application of these membranes in environments requiring stringent antibacterial properties, with future research focusing on their long-term stability, reusability, and broader spectrum efficacy. It should be noted that the CFU counts reported here represent single-point measurements without statistical replication due to experimental constraints. While standard deviations are not provided, the observed trends are consistent and qualitatively support the antibacterial effect of the modified membranes. Future work will include full statistical analysis to validate these findings.

## 4. Conclusions

This study successfully developed four straightforward and easily scalable methods to modify dicarboxylated polysulfone membranes, including crosslinking with branched polyethylenimine and ethylenediamine (method A), partial hydrolysis in PSF-EDA to form tailored voids and aminated brushes (method B), attachment of virous alkyl brushes (method C), and formation of quaternary ammonium motifs on membranes (method D).

Table 2 presents a comparative overview of the four modification approaches, highlighting their structural differences and their key effects on membrane. The modifications yielded novel membranes with enhanced CaCl_2_ rejection compared to the unmodified PSF-COOH membrane, effectively addressing its inherent limitations. Among the developed membranes, PSF-PEI exhibited the highest permeance while maintaining effective salt rejection. In contrast, PSF-EDA showed reduced permeance due to increased crosslinking density. However, partial hydrolysis of the crosslinked network in PSF-EDA resulted in PSF-NH_2_, which exhibited increased permeance without a significant loss in rejection. Introducing longer polymer brushes in PSF-NH_2_ would likely further reduce permeance and increase salt rejection, as observed in PSF-C12, which demonstrated low permeance and improved ion exclusion.

Although the performance of the modified membranes developed in this study does not yet match that of commercial nanofiltration membranes in terms of salt rejection and permeance, the methods presented offer a straightforward and modular approach for further functionalization of carboxylated membranes, expanding their potential applications. Membranes functionalized with amine groups, such as PSF-PEI and PSF-NH_2_, show promise for CO_2_ separation applications. Membranes attached with aliphatic brushes can be used in the separation of hydrocarbons in organic phases. Meanwhile, quaternary ammonium-functionalized membranes, such as QA-PEI-PSF and QA-NH_2_-PSF, could be further optimized for use in antibacterial filtration systems.

## Figures and Tables

**Table 1:** Bacterial growth on different membranes.

Membrane Type	Bacterial Species	CFUs (0 min)	CFUs (90 min)	Relative Change (%)
Parafilm	*E. coli*	3	150	4900%
PSF	*E. coli*	20	259	1195%
PSF	*Bacillus*	19	88	363%
QA-PEI-PSF	*E. coli*	257	170	−34%
QA-PEI-PSF	*Bacillus.*	8	0	−100%
QA-NH_2_-PSF	*E. coli*	190	91	−52%
QA-NH_2_-PSF	*Bacillus*	4	2	−50%

Note: Relative Change (%): Percentage change in CFU count from 0 min to 90 min for each membrane.

**Table 2:** Summary of four modification methods and their performance.

Method	Functionalization	Key Effect on Membrane
Method A	PEI or EDA crosslinking	Improved salt rejection (CaCl_2_); moderate permeances
Method B	Partial hydrolysis of PSF-EDA and formed designed voids and brushed	Improved permeance and maintained salt rejection compared with PSF-EDA
Method C	Alkyl brushes attaching	Lower permeance and higher rejection with longer brushes
Method D	Quaternary ammonium	Biofilm prevention effect and improved CaCl_2_ rejection; low permeance

## Data Availability

The data presented in this study are available on request from the corresponding author. The data are not publicly available due to privacy reasons.
